# SeqDoC: rapid SNP and mutation detection by direct comparison of DNA sequence chromatograms

**DOI:** 10.1186/1471-2105-6-133

**Published:** 2005-05-31

**Authors:** Mark L Crowe

**Affiliations:** 1 Institute for Molecular Bioscience, The University of Queensland, Brisbane, Queensland 4072, Australia; 2 The Australian Research Council Special Research Centre for Functional and Applied Genomics, The University of Queensland, Brisbane, Queensland 4072, Australia

## Abstract

**Background:**

This paper describes SeqDoC, a simple, web-based tool to carry out direct comparison of ABI sequence chromatograms. This allows the rapid identification of single nucleotide polymorphisms (SNPs) and point mutations without the need to install or learn more complicated analysis software.

**Results:**

SeqDoC produces a subtracted trace showing differences between a reference and test chromatogram, and is optimised to emphasise those characteristic of single base changes. It automatically aligns sequences, and produces straightforward graphical output. The use of direct comparison of the sequence chromatograms means that artefacts introduced by automatic base-calling software are avoided. Homozygous and heterozygous substitutions and insertion/deletion events are all readily identified. SeqDoC successfully highlights nucleotide changes missed by the Staden package 'tracediff' program.

**Conclusion:**

SeqDoC is ideal for small-scale SNP identification, for identification of changes in random mutagenesis screens, and for verification of PCR amplification fidelity. Differences are highlighted, not interpreted, allowing the investigator to make the ultimate decision on the nature of the change.

## Background

The ability to identify single nucleotide changes in DNA is a fundamental requirement in many fields of biological research. The identification and characterisation of naturally-occurring single nucleotide polymorphisms (SNPs) underlies a vast body of work on genetically-linked disorders, diagnosis and risk prediction [1-4] as well as being important in genomic mapping and population genetics [5-8]. Identification of point mutations is of equal importance to many researchers, for roles as diverse as identifying specific alterations caused by random mutagenesis screens [9,10] to validation of the fidelity of sequences amplified by PCR.

For labs studying SNPs or point mutations, identification of these can be a time-consuming and error-prone process, particularly if novel changes are being investigated. In some cases, software such as the Staden package [11,12] or Sequencher [13] may provide a suitable solution. However these are sophisticated and multifunctional programs, and can prove overly complex for simple sequence comparisons. Consequently, many small-scale projects may rely solely on manual analysis, for example simply carrying out a direct text comparison of the processed sequence to a known reference.

This manual approach is affected by variations in sequence quality and incorrect base calling, and may also miss heterozygous bases if, for example, the wild-type peak is higher that the additional peak. To address these issues and to provide a simple and efficient way to accurately identify sequence changes, we have developed a web-based application which compares DNA sequence chromatograms directly. SeqDoC (Sequence Difference of Chromatograms) is freely accessible, very easy to use, and highlights differences characteristic of single base changes, including heterozygous SNPs and insertions and deletions.

## Implementation

### Read in chromatograms

Two ABI sequence chromatograms, one a reference and the other the test sequence, are the only user-supplied data. These are uploaded through a web form and the sequence traces and other relevant data extracted using the Perl ABI.pm module [14]. The sequence traces for each channel (i.e. A, C, G and T) are stored as individual arrays within the chromatogram object. Blank sequence at the beginning and end of each chromatogram (resulting for example from sequencing of a PCR product, when the trace continues past the end of the template) are removed by deleting the terminal values from the traces where all channel values are less than 50. In tests, we established that a filter value of as low as five resulted in the removal most blank terminal sequence, while a value of as high as 500 still retained virtually all genuine sequence; we therefore used 50 as an appropriate intermediate value.

### Normalize traces

Comparison of the test to the reference sequence is performed by subtraction of trace values, so it is necessary to normalize the trace values so that a sequence run with strong signal can be meaningfully compared to one with weaker signal. Normalisation is performed for each channel individually, and scales each data point so that the local mean value for that channel is 100 (local being defined as 500 data points prior to the point being scaled, the point itself and 499 points after). The mean value of those local points for the channel is calculated and divided by 100 to give a scaling factor, and the point being normalized is then scaled by being divided by this factor.

Special exceptions are made for the initial 500 and final 499 values of the trace, where there are either not 500 values before, or not 499 after, the point being normalized. For these two cases, the mean is based on as many points as are present between the point and the end of the sequence on one side, while still using 500 on the other side.

### Align traces

Due to variations arising both from the sample and the sequencing matrix, the start position of the sequence traces and the base separation within the traces may differ between the test and the reference sample. The software compensates for this by automatically calculating the optimal start alignment combined with continual fine adjustment throughout the length of the sequences to maintain alignment of the test and reference sample.

To identify start point offsets, the software tests a range of initial alignments, from -200 to +200 data points, corresponding to approximately +/- 20 bases of sequence. The offset which results in the best alignment (the smallest total value of the absolute differences between the test and reference traces for all four channels) is used for subsequent analysis. This is implemented by the addition or deletion of 'spacer' data points at the beginning of the test sequence.

Ongoing fine adjustment of the sequences is achieved by the addition or removal of individual data points from the test sequence as required. The sequences are sampled every five data points, and difference scores calculated for the subsequent 30 data points. If that difference score is reduced by the insertion or deletion of a single data point, then the test traces are adjusted accordingly (by either duplication or removal of the data point at the test position).

### Calculate differences

Following normalisation and alignment of the sequences, a 'difference profile' is calculated. The trace values of the test sequence are subtracted from the equivalent values for the reference sequence for all four channels, and the resulting values are passed through an algorithm which highlights changes characteristic of base substitutions, while reducing random noise. This is achieved by squaring the difference value and multiplying the result by the square root of the sum of the differences of other channels which change in the opposite direction.

The overall outcome of this process is to enhance the signal given by large differences with at least one channel changing in the opposite direction, which is characteristic of base substitutions, while minimising the signal from small unidirectional changes (typical of signal noise). Difference profiles are calculated for all four channels and the results overlaid in the final output.

### Generate trace and difference images

User output is provided in the form of three aligned images: aligned sequence chromatograms for the reference and test sequences, and a similarly aligned difference profile. These outputs are based on the normalized, aligned traces generated during earlier stages of the analysis. The difference trace is typically primarily flat, with a large bidirectional peak at the point of any base changes between the sequence. Other patterns are possible, depending on specific features of the test and reference sequences, and are discussed in more detail below. The three images are generated by the Perl GD::Graph module, and are returned to the user as a web page. Identification of the site of base substitutions is simply a matter of examining the difference trace for the bidirectional peaks mentioned above; by examining the aligned test and reference sequences at the point of these peaks, single base changes between the two sequences can be rapidly and simply identified.

### Staden comparison

The Staden 1.5.3 Windows installer was downloaded from SourceForge and installed on a PC running Windows XP Pro. Tracediff comparison was performed through Pregap4 using the following modules in order: General Configuration, Initialise Experiment Files, Reference Traces & Sequences, Trace Difference. All user-definable parameters were left at their default values (except that 'Write trace differences out to disk' was selected). We used Gap4 to both align and view the initial and tracediff-generated traces as well as to carry out trace subtraction directly. The reference and test sequences (and difference trace where appropriate) were assembled into a contig, which was opened in the Trace display window. A subtracted trace was generated using the Diff button.

## Results and discussion

All scaling factors, cutoff filters and algorithms described in the methods section were chosen after testing of multiple settings as giving the clearest identification of single base changes and best retention of genuine data while minimising the signal resulting from noise. The process was initially optimised using sequences covering known polymorphisms in different regions of the human melanocortin 1 receptor gene [15]. In all cases the polymorphisms were clearly visible in the difference trace. The software has since been successfully used to test for single base changes in several hundred sequence chromatograms.

**Figure 1:**
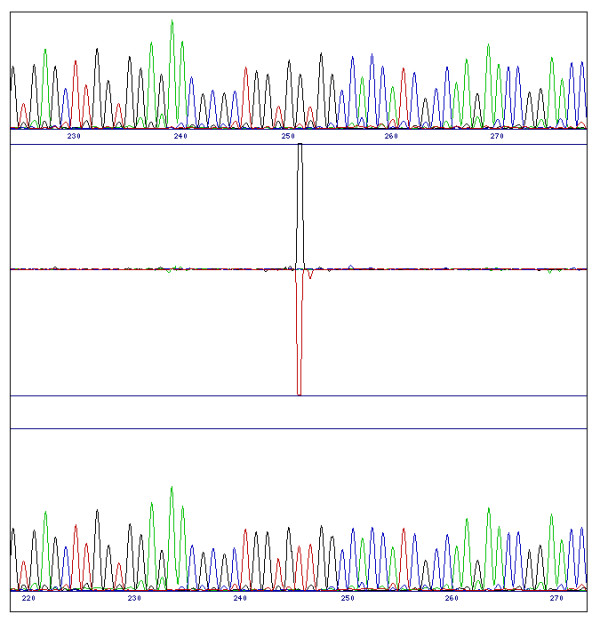
**Difference pattern from a single base substitution**. Replacement of one base by another (here a G for an A at position 251 in the reference sequence (top trace)) means a major decrease in the value of one channel and a similar increase in another. This causes a bi-directional peak in the difference profile.

**Figure 2:**
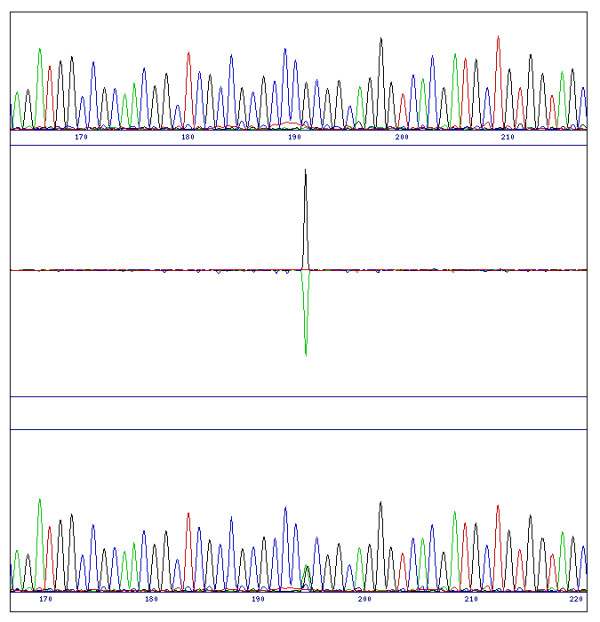
**Difference pattern from a heterozygous SNP**. A multiple peak in the test sequence (bottom trace) is characterised by an increase in the value in one channel (in this case the A at position 195 in the test sequence), and typically a decrease in the original channel (G), therefore giving a bi-directional peak in the difference profile similar to a direct substitution.

Extracts from typical output traces are shown in figures 1 and 2, which identify homozygous and heterozygous polymorphisms respectively. The difference trace does not differentiate between these two different substitutions (although the size of the double peak is typically smaller for heterozygous sites). Instead it makes it rapidly obvious to an investigator where the sites of difference are, and allows the investigator to make the final decision about the nature of the substitution.

**Figure 3:**
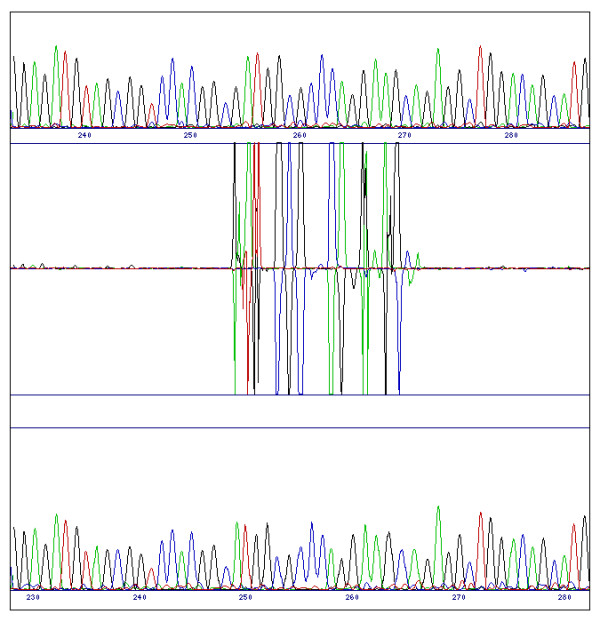
**Difference pattern from a single base deletion**. When a base is deleted (in this case the G at position 254 in the reference sequence) the resulting phase shift in the test sequence will result in major differences between the traces until the software compensates by bringing them back into alignment.

SeqDoC is also able to highlight the occurrence of single base insertion or deletion events. Figure 3 shows the typical pattern from a deletion; at the point of the deletion, there is a major difference between the two chromatograms, which is gradually eliminated by the alignment algorithm bringing the two chromatograms back into phase. Insertion events give similar patterns. As with substitutions, no interpretation of the pattern is calculated; the role of the software is to alert the investigator to the location of the change, not to characterise it.

The automatic start alignment process means that it should not be necessary to use the same primer to sequence the reference and test sequences, providing that the two different primers produce sequences which start within approximately 20 nucleotides of each other. Alternatively, it would be possible to manually edit the raw chromatograms to bring the start points of the two sequences into approximate alignment. We do not believe that the comparison will work for samples sequenced in opposite directions, using the reverse complement of one or the other. Sequencing chemistry is such that peak heights are typically affected by the local sequence composition [16,17], and while this is consistent for samples sequenced in the same direction from different primers, it is not true for those sequenced in opposite direction.

Because of the normalisation and noise reduction algorithms built into the software, it is relatively resilient to poor quality sequence. Typically, if the sequence quality is sufficient for an investigator to unambiguously identify the base call, it is good enough for automatic identification of sequence differences. Most problems with sequence quality only occur at the end of the sequence run, although unincorporated dye terminators may cause 'dye blobs' at the beginning of the sequence, which can partially mask base changes occurring at the same site. Examples of the output produced in these cases, along with full instructions on the use of SeqDoC, are provided on our website at .

The Staden software package [11] is an established, well-supported and widely used sequence analysis package, and has functions (such as 'tracediff') for direct comparison of chromatogram traces analogous to those provided by SeqDoC. It can also display trace subtractions through the Gap4 program. We have therefore evaluated the performance of SeqDoC using Staden as a benchmark. Although the principle advantage of SeqDoC over Staden is that it is specifically designed and optimized for the single purpose of chromatogram comparison, and therefore provides a much simpler user interface, we also believe that it offers advantages in normalisation, alignment and, particularly, sensitivity. On the other hand, Staden can allow much higher throughput, since multiple sequences can be analysed at once; data can also be saved and analysed in detail with multiple additional functions.

**Figure 4:**
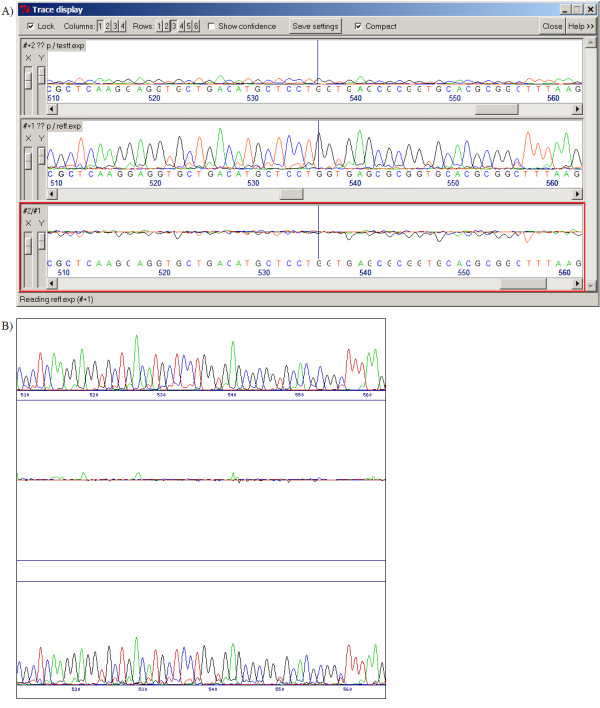
**Comparison of weak and strong sequence traces**. Weak and strong sequence traces are successfully aligned by both Staden's Gap4 program (fig 4a) and SeqDoC (fig 4b). The local normalisation algorithms of SeqDoC mean that the two traces are displayed with comparable peak heights (weak trace at the top), and possibly results in a less noisy difference profile. Both figures show the same region of the same sequences.

**Figure 5:**
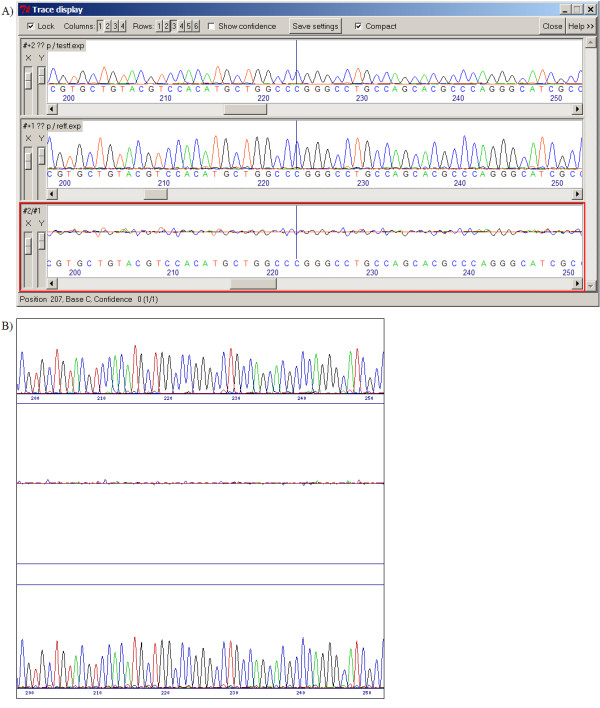
**Effects of sequence misalignment**. A minor misalignment of test and reference sequences by Gap4 causes a cyclical pattern in the difference profile (fig 5a, bottom trace) which increases signal noise. The fine adjustment algorithm of SeqDoC ensures that sequences are properly aligned (fig 5b) and eliminates this noise signal.

Figure 4 shows a comparison of a weak and strong signal trace with Staden and SeqDoC respectively. The SeqDoC local normalisation algorithm means that the trace heights for both are very similar, and therefore more readily comparable. Although the Y-scale can be altered in the Staden trace display window to compensate for this, the problem is then shifted to the beginning of the trace where the sequence is proportionally much stronger. The effects of misalignment are observed earlier in the same comparison, where the Staden difference trace shows a characteristic cyclical pattern which is not observed in the SeqDoC alignment (figure 5). Although it is impossible to tell whether either of these factors significantly compromises the performance of tracediff, we suspect that alignment at least will have some influence, since any misalignments will introduce unnecessary noise into the difference trace.

**Figure 6:**
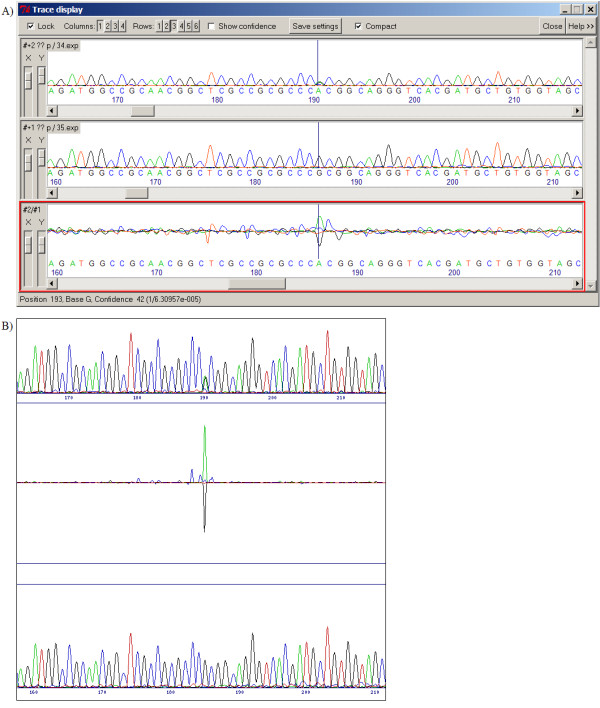
**Missed heterozygous base call**. Tracediff does not identify the heterozygous base at position 191 in the test sequence (upper trace, fig 6a), possibly because of the weak signal strength and noise in the difference profile. The normalisation and difference profile optimisation algorithms built into SeqDoC give a very strong signal to noise ratio for this change in the difference profile (fig 6b) and make the substitution obvious.

**Figure 7:**
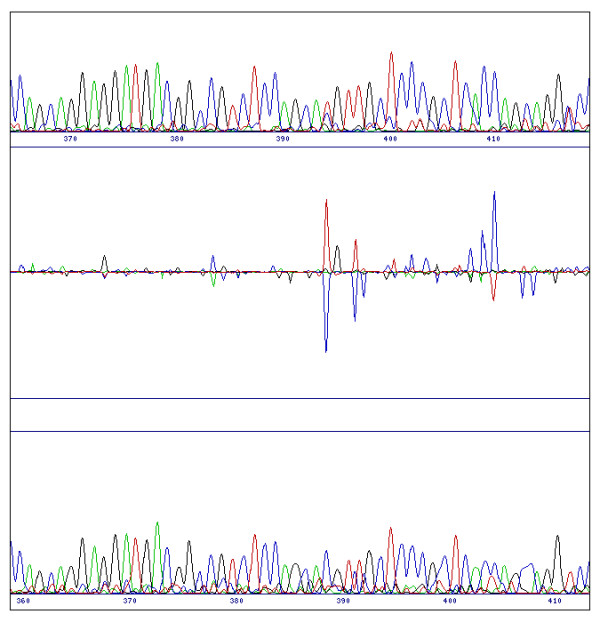
**Poor sequence quality**. A poor quality sequence (in this case the reference sequence, top) unavoidably causes a noisy difference profile. However SeqDoC still successfully highlights nucleotides which differ between the sequences. In this case the C at position 390 in the reference sequence becomes a mixed T/C peak at position 398 in the test sequence.

The main functional benefit of using SeqDoC over Staden is that of sensitivity, particularly for identifying heterozygous peaks or when using either weak or poor quality sequence. The heterozygous base shown in figure 2 is identified by tracediff, but the output suggests that it is a direct replacement rather than a mixed base, while another (figure 6) is missed by tracediff altogether. The latter example occurs in a weak strength trace, which is compensated for by the SeqDoC normalisation. Similarly tracediff can miss differences in noisy sequence; SeqDoC is more robust, because calls are made by visual inspection and the difference profile is used only to draw the investigator's attention to areas of difference. For example, figure 7 shows a comparison using poor quality reference sequence data. Although the difference trace is consequently noisy, it still highlights a heterozygous substitution in the test sequence.

In summary, SeqDoC proves a lightweight but effective substitute to Staden for sequence trace comparisons. While Staden is a more appropriate choice for applications where high throughput is the main priority, SeqDoC provides a better solution when sensitivity, specificity or simplicity are more important considerations.

## Conclusion

SeqDoC is a very easy to use web-based application which rapidly highlights differences between ABI sequence chromatograms, including substitution and insertion/deletion events. It uses chromatograms directly, rather than extracted text-based sequence data, so eliminating errors introduced by base calling software and allowing identification of heterozygous substitutions which might otherwise be missed. No user intervention or adjustment is required for processing, with all normalisation, alignment and noise reduction being carried out automatically; on the other hand the ultimate decision on the specific change identified remains with the investigator. SeqDoC is free and requires no training to use, and is ideally suited for use by researchers carrying out small scale SNP analysis or mutagenesis experiments. It can also be used to rapidly screen PCR-amplified products for introduced mutations.

## Availability and requirements

Program name: SeqDoC

Project home page: 

Source code:  or additional file 1.

Operating system(s): Platform independent

Programming language: Perl CGI

Other requirements: Requires Perl CGI, GD::Graph and ABI modules

License: None for web access, GNU GPL for source code

Any restrictions to use by non-academics: No restrictions

## Supplementary Material

Additional File 1 The perl source code for SeqDoC (seqdoc.pl) is available with the online version of this article (additional file 1). Instructions for use of the program can be obtained using the 'perldoc' command. Click here for file
